# The Role of Surface Topography in Bacterial Adhesion to Amorphous TiO_2_-Coated PMMA

**DOI:** 10.3390/dj14080463

**Published:** 2026-07-24

**Authors:** Iva Zeneral Žuža, Iris Car Kubaska, Krunoslav Bojanić, Ivana Jelovica Badovinac, Zoran Kovač, Marko Perčić, Robert Peter, Iva Šarić Janković, Maria Kolympadi Marković, Sandra Kraljević Pavelić, Dean Marković

**Affiliations:** 1 Department of Oral Surgery, Faculty of Dental Medicine, University of Rijeka, 51000 Rijeka, Croatia; iva.zeneral.zuza@fdmri.uniri.hr; 2 Faculty of Biotechnology and Drug Development, University of Rijeka, 51000 Rijeka, Croatia; iris.car.kubaska@uniri.hr; 3 Laboratory for Aquaculture Biotechnology, Division of Materials Chemistry, Ruđer Bošković Institute, 10000 Zagreb, Croatia; kbojanic@irb.h; 4 Faculty of Physics, University of Rijeka, 51000 Rijeka, Croatia; ijelov@phy.uniri.hr (I.J.B.); rpeter@phy.uniri.hr (R.P.); iva.saric@phy.uniri.hr (I.Š.J.); maria.kolympadi@phy.uniri.hr (M.K.M.); 5 Centre for Micro- and Nanosciences and Technologies, University of Rijeka, 51000 Rijeka, Croatia; marko.percic@uniri.hr; 6 Department of Prosthodontics, Faculty of Dental Medicine, University of Rijeka, 51000 Rijeka, Croatia; zoran.kovac@fdmri.uniri.hr; 7 Faculty of Engineering, University of Rijeka, 51000 Rijeka, Croatia; 8 Faculty of Health Studies, University of Rijeka, 51000 Rijeka, Croatia

**Keywords:** polymethyl methacrylate, atomic layer deposition, titanium dioxide coating, surface roughness, surface topography, bacterial adhesion, biofilm formation, UV irradiation

## Abstract

**Background/Objectives:** Polymethyl methacrylate (PMMA) is widely used in medical and dental applications because of its favorable mechanical properties and ease of processing. However, its clinical performance is limited by low surface hardness, hydrophobicity, and susceptibility to microbial colonization and biofilm formation. This study aimed to investigate the effects of amorphous titanium dioxide (TiO_2_) nanolayers deposited by atomic layer deposition (ALD) on the surface morphology and antibacterial properties of PMMA-based materials. **Methods:** Amorphous TiO_2_ coatings were deposited on bone cement PMMA and dental PMMA substrates using ALD with TiCl_4_ and H_2_O precursors at 80 °C. The low deposition temperature enabled the conformal of thermally sensitive polymer substrates. Surface characterization was performed using atomic force microscopy (AFM) and scanning electron microscopy (SEM) to evaluate coating morphology and nanoscale topography. Antibacterial activity was assessed against *S. aureus* and *P. aeruginosa* through planktonic growth and biofilm formation assays, with additional evaluation of ultraviolet (UV) activation and surface polishing. **Results:** AFM analysis revealed that amorphous TiO_2_ coating on standardly laboratory practice-polished PMMA increased the arithmetical mean roughness (Ra) from 1.82 nm to 14.60 nm, and maximum height (Rmax) from 36.20 nm to 298.00 nm. Polishing before coating significantly increased surface roughness and height variation, resulting in complex micro- and nanotopography. Microbiological analyses demonstrated variable antibacterial effects depending on bacterial species and surface characteristics. Comparison of planktonic growth, biofilm formation and OD590 ratio showed that amorphous TiO2 coating on polished PMMA reduced biofilm formation and planktonic growth in *P. aeruginosa* with a decreased OD ratio, while *S. aureus* biofilm formation was reduced. *S. aureus* had a consistently higher OD590 than *P. aeruginosa*. UV treatment alone did not produce consistent antibacterial enhancement. **Conclusions:** The study findings suggest that the surface topography had a greater role than UV treatment in determining bacterial adhesion. Surface roughness was strongly associated with *S. aureus* adhesion., whereas *P. aeruginosa* showed minimal response to the tested surface modifications. These findings suggest that TiO_2_ coating after standard polishing alone may not provide consistent antibacterial activity under the tested conditions and point to the importance of nanoscale surface design in developing antimicrobial polymer biomaterials.

## 1. Introduction

Titanium dioxide is an inorganic compound widely used in biomedical applications, especially in dental medicine, due to its high biocompatibility, chemical stability and corrosion resistance [1,2,3]. In addition to its favorable biological properties such as promotion of cell adhesion, proliferation and mineralization, TiO_2_ exhibits photocatalytic activity under UV irradiation, generating reactive oxygen species (ROS) that may contribute to antibacterial effects and biofilm control on material surfaces [4,5]. Besides its antimicrobial effects it is used as a drug delivery carrier, enabling the release of therapeutic drugs such as antibiotics [6]. TiO_2_ can be deposited on a broad spectrum of substrates, including metals, polymers, ceramics and glass, using various depositing techniques such as anodization, micro-arc oxidation, sol–gel processing, vapor and laser deposition and atomic layer deposition (ALD). Among these, ALD, which uses gas precursors reacting alternatively on surface groups of solid substrates, has emerged as a key technique. It enables the formation of highly uniform, conformal thin films on complex three-dimensional geometries with atomic-scale thickness control at relatively low processing temperatures [7].

Although ALD has been predominantly applied to metallic substrates such as dental implants and alloys, increased attention has been directed toward its application on polymeric materials, particularly polymethyl methacrylate (PMMA). PMMA is used in dental medicine as a standard material for provisional restorations, bridges, and denture bases due to its transparency and ease of processing, while in medicine it is used as a biomaterial for bone cements, intraocular and contact lenses, orthopedic fixation, filling bone defects and cranial cavities, as well as for vertebral stabilization procedures such as vertebroplasty in osteoporotic patients [5]. However, its clinical application is limited by inherent drawbacks such as low surface hardness, poor wear resistance, porosity, residual monomer release, pronounced hydrophobicity, and a high susceptibility to microbial colonization, all of which may compromise its long-term performance [2,5]. Microbial colonization of PMMA-based restorations and prostheses is still a major clinical concern as biofilm accumulation can contribute to many oral cavity problems, i.e., denture stomatitis, secondary infections, unpleasant odor, discoloration, and material deterioration/reduced longevity of dental prostheses. Therefore, developing durable surface modification strategies that reduce bacterial adhesion without compromising the bulk properties of PMMA remains an important goal in biomaterials research. In this context, surface topography, rather than chemical composition alone, is increasingly recognized as a critical determinant of bacterial adhesion. These limitations have driven the development of surface modification strategies, including the deposition of TiO_2_ nanolayers to improve both mechanical and biological performance. The relative contribution of ALD-induced nanoscale topography versus photocatalytic activity in polymer systems, however remains unclear [8]. Although numerous studies have demonstrated antibacterial effects of TiO_2_ coatings, these effects are frequently attributed to photocatalytic activity and are not distinguished from changes in surface morphology after coating deposition. It accordingly remains unclear whether reduced bacterial adhesion on TiO_2_-coated PMMA results primarily from photocatalytic mechanisms or from ALD-induced modifications of nanoscale surface topography.

A major challenge in applying ALD to PMMA is the lack of reactive surface groups required for precursor adsorption and film nucleation [9]. To overcome this limitation, surface activation methods such as plasma treatment, UV irradiation, and chemical functionalization have been employed to introduce reactive sites [10]. In addition, plasma-enhanced ALD (PEALD) enables deposition at lower temperatures, minimizing polymer degradation while maintaining coating quality. Although TiO_2_ can be incorporated into PMMA or applied as a surface coating, coating approaches generally demonstrate superior antimicrobial efficacy [11,12]. Some studies suggest that adding TiO_2_ nanoparticles may reduce the natural porosity of PMMA, which is clinically relevant because surface pores and irregularities allow microorganisms to adhere and penetrate the material [13].

The present study focuses on the role of surface modification in TiO_2_-coated PMMA. Rather than approaching TiO_2_ solely as an antibacterial agent, this work investigates how ALD alters surface morphology and how these topographical changes govern material–bacteria interactions [14]. The combined use of SEM and AFM enables correlation between surface morphology and quantitative roughness parameters, providing a multiscale understanding of how surface features influence bacterial attachment. This integrated approach may provide insights into whether the antibacterial performance of TiO_2_-coated PMMA is primarily governed by surface topography, thereby supporting the rational design of antimicrobial polymer biomaterials. Amorphous TiO_2_ thin films which can be deposited easily by ALD at low temperatures are compatible with sensitive substrates, such as polymers. Amorphous TiO_2_ has been shown to be photocatalytically active upon illumination with UV light, but it is less efficient compared to crystalline anatase TiO_2_ obtained during high deposition temperatures (>200 °C) or by post-preparation annealing [15]. However, previous studies have shown the photocatalytical bacterial growth inhibition activities of amorphous TiO_2_ [16,17,18]. Based on these, we have decided to investigate the various parameters that influence the antibacterial activities of amorphous ALD TiO_2_ coatings on polished PMMA as deposited or under photocatalysis with the view of applications in dental medicine practice.

In the presented study, we hypothesized that low-temperature ALD of amorphous TiO_2_ on polished PMMA would modify the nanoscale surface topography while forming a uniform coating, thereby reducing bacterial adhesion independently of UV-induced photocatalytic activation.

## 2. Materials and Methods

### 2.1. Sample Preparation

PMMA specimens were fabricated from commercially available bone cement (Cemex System, Tecres S.p.A., Orthopaedic Bone Cement, Verona, Italy) and highly cross-linked dental PMMA (Ivoclar Vivadent AG, Schaan, Liechtenstein). The dental PMMA blocks were polymerized under high temperature and pressure according to the manufacturer’s protocol. From these standard PMMA blocks, square specimens measuring 5 × 5 × 2 mm were sectioned using computer-aided design and computer-aided manufacturing (CAD/CAM). The specimen preparation was performed in the dental laboratory Dentex 3D d.o.o., Rijeka, Croatia. The samples were divided into experimental groups according to the type of PMMA material and surface modification. Bone cement specimens were prepared either as unmodified controls or coated with a TiO_2_ nanolayer. These samples were used to evaluate the effect of the TiO_2_ coating. Dental PMMA specimens were grouped into separate experimental series, each designed to investigate a specific parameter. Depending on the experimental design, comparisons included surface finishing (polished vs. unpolished), synthesis temperature (80 °C vs. 230 °C), or ultraviolet (UV) irradiation (UV-exposed vs. non-irradiated). Results for the 230 °C deposition condition are presented in the Supplementary Materials Tables S1–S9. For experiments involving surface finishing, polishing was performed in a certified dental laboratory according to standardized protocols for PMMA-based materials to ensure consistent and reproducible surface characteristics across all samples. These variables were not applied simultaneously in all experimental series but were evaluated independently to determine their individual effects on surface properties and antimicrobial behavior.

For each experimental series, all specimens were prepared in the same batch under identical fabrication conditions. Following preparation, representative specimens from each experimental group were allocated for AFM characterization, whereas separate specimens from the same batch were used for microbiological analyses.

### 2.2. Atomic Layer Deposition (ALD)

TiO_2_ thin films were deposited on previously prepared PMMA specimens using ALD. The process was carried out in a Beneq TFS 200 system (Beneq Oy, Espoo, Finland), using titanium tetrachloride (TiCl_4_, 99.9%, Sigma-Aldrich, St. Louis, MO, USA) as the metal precursor, water vapor as the oxidizing agent, and ultra-high purity nitrogen (N_2_, 6.0) as both carrier and purging gas. Deposition was performed at two temperatures, 80 °C and 230 °C, in order to investigate the influence of film structure on material properties, specifically the formation of amorphous versus crystalline TiO_2_ phases. Each ALD cycle consisted of 250 ms exposure to TiCl_4_, followed by a 6 s of N_2_ purging, a 180 ms pulse of water vapor, and a 4 s N_2_ purge. The 100 nm thick films were deposited using 1500 ALD cycles, the 70 nm thick films using 1050 ALD cycles, and the 30 nm thick films using 450 ALD cycles. The 30 nm coating served as the lower-thickness reference for morphological comparison, whereas the 70 nm and 100 nm coatings were selected for microbiological experiments to evaluate antibacterial performance of representative thicker TiO_2_ coatings.

### 2.3. Atomic Force Microscopy (AFM) Analysis

The surface topography of the PMMA specimens was characterized by AFM using a Dimension Icon system (Bruker Corporation, Billerica, MA, USA) operating in dynamic mode. Three groups of samples were analyzed: unpolished PMMA, polished PMMA, and polished PMMA coated with amorphous TiO_2_ thin film of 100 nm thickness deposited by the ALD method as described above. Imaging was performed using a Bruker SNL-10 Type D silicon nitride cantilever with a silicon tip and a nominal tip radius of 6 nm (Bruker Corporation, Billerica, MA, USA). The cantilever was moderated by the method of thermal oscillations to determine its dynamic and mechanical properties. Scans were carried out over an area of 1 μm^2^ with a resolution of 512 × 512 points, with three repeated measurements made for each sample. The obtained data were processed in the Bruker Nanoscope Analysis program with tilt and bow correction. Standard roughness parameters (Ra, Rq and Rmax) were calculated to quantitatively assess changes in surface morphology after polishing and deposition of amorphous TiO_2_ thin film.

### 2.4. Scanning Electron Microscopy (SEM) Analysis of PMMA Surfaces

The surface morphology of PMMA specimens and amorphous TiO_2_ thin films of 100 nm thickness deposited by the ALD method was examined by SEM using a JEOL JSM-7800F microscope (JEOL Ltd., Tokyo, Japan) at the Joint Laboratory of the Faculty of Physics and the Centre for Micro- and Nanosciences and Technologies, University of Rijeka. Prior to microbiological experiments, SEM imaging was performed to evaluate the surface morphology of the PMMA substrates and the homogeneity of the amorphous TiO_2_ coating. The specimens were coated with a thin conductive Au/Pd layer using sputter coating to prevent surface charging and mounted on aluminum stubs using conductive carbon tape. Imaging was carried out at an accelerating voltage of 5–10 kV (10 kV for samples without bacteria and 8 kV for samples with bacteria). SE imaging was used to evaluate surface topography, while BSE imaging was employed to determine the thickness of the ALD-deposited TiO_2_ films by analyzing their cross-section using secondary electron (SE) (JEOL Ltd., Tokyo, Japan) and backscattered electron (BSE) detectors. After microbiological testing, SEM was additionally used to analyze bacterial biofilm formation. One specimen representing a technical replicate was selected from each well. For each specimen, six SEM images were acquired: one image at 1000× magnification to assess the overall distribution of the biofilm on the surface, and five images at 5000× magnification obtained from different randomly selected areas of the same specimen to visualize adherent bacterial cells and their organization in greater detail.

### 2.5. Microbiological Analysis and UV Irradiation of PMMA Specimens

#### 2.5.1. Bacterial Strains and Inoculum Preparation

Microbiological experiments were performed using the reference strains *Staphylococcus aureus* ATCC 25923 and *Pseudomonas aeruginosa* ATCC 27853. Biofilm formation tests were performed according to recommendations for assays based on microtiter plates [19] adapted for the testing of hard materials [20]. Briefly, bacterial cultures were grown on tryptic soy agar (Biolife Italiana S.r.l., Milan, Italy) from cryovials stored at −80 °C and sub-cultured to obtain fresh exponential growth of F2 or F3 generation for experimental runs. The bacterial inoculum was prepared in tryptic soy broth (TSB) supplemented with 1% (*w*/*v*) glucose, standardized to 0.5 McFarland (≈1.5 × 10^8^ CFU/mL) by turbidity adjustment (bioMerieux Inc., Marcy-l’Étoile, France), and diluted to a final concentration of 5 × 10^5^ CFU/mL. Optical density of bacterial suspensions and planktonic growth was verified at 600 nm (OD600) using a UV/VIS spectrophotometer (Infinite 200 PRO, Tecan Group Ltd., Grödig, Austria).

#### 2.5.2. Biofilm Formation Assay

Prior to inoculation, TiO_2_-coated PMMA samples were disinfected by immersion in 96% ethanol for 30 min and dried under sterile conditions. Three milliliters of bacterial suspension were added to each well of a 6-well plate (Thermo Fischer Scientific, Waltham, MA, USA) to ensure complete immersion of the samples. Negative control wells contained sterile medium without inoculum and samples (sterility of media evaluated), and positive controls contained medium and inoculum without samples (growth of bacteria in media evaluated). Samples were incubated for 24 h at 37 °C under static aerobic conditions. After incubation, samples were rinsed with phosphate buffer to remove non-adherent and loosely attached bacteria. Preliminary tests of PMMA coating effect were performed using three technical and three analytical replicates for all growth and biofilm indices followed. Final experiments were performed in three independent biological replicates, each including three technical replicates per condition. The antimicrobial performance of TiO_2_-coated PMMA was evaluated by assessing planktonic bacterial growth and surface-associated biofilm formation. Biofilm-associated cells were detached from the sample surface by gentle pipetting and vortexing, resuspended in phosphate buffer, and quantified spectrophotometrically at OD600, and were serially 10-fold diluted for CFU counting.

#### 2.5.3. Crystal Violet Biofilm Quantification

Total biofilm biomass on PMMA surfaces was also determined using the crystal violet assay. Biofilm was heat-fixated at 60 °C for one hour, washed again, and stained with 0.1% (*v*/*v*) crystal violet for 15 min. Samples were then rinsed with phosphate buffer, dried and immersed in 96% ethanol for 30 min to resolubilize the bound crystal violet. A 100 µL aliquot of the obtained solution was transferred to a 96-well plate, and absorbance was measured at 590 nm (OD590). Biofilm formation by OD ratio was determined by dividing the OD590 of samples by the OD590 cut-off value of sample negative controls (sample with sterile media) set by adding 3 standard deviations to the average value. The sample negative control serves to correct for background non-specific staining of crystal violet. For qualitative assessment, samples with OD ratio > 1 are considered positive for biofilm formation and ≤1 as negative, i.e., no biofilm was formed. Quantification of biofilm formation was as follows: OD ratio of 0–1 no biofilm, 1–2 weak biofilm, 2–4 moderate biofilm, and >4 strong biofilm formed. Measurement variability was assessed using standard deviation (SD).

#### 2.5.4. Experimental Design and UV Irradiation

Four independent experiments were conducted to systematically evaluate factors influencing the antimicrobial behavior of amorphous TiO_2_-coated PMMA. First, unmodified bone cement PMMA was compared with TiO_2_-coated bone cement PMMA to determine the baseline effect of the coating. Second, the influence of deposition temperature was investigated using dental PMMA samples coated by ALD at 80 °C and 230 °C. Third, photocatalytic activation was examined by irradiating TiO_2_-coated dental PMMA (80 °C) with ultraviolet light at 254 nm and 366 nm for 10 min and 1 h (~2 mW/cm^2^). Finally, the combined influence of surface finishing and UV activation was evaluated by comparing unpolished, polished, and polished UV-treated TiO_2_-coated PMMA samples. UV irradiation was performed using a CAMAG^®^ UV Lamp 4 dual-wavelength system (CAMAG AG & Co., Muttenz, Switzerland). In the initial experiments, samples were irradiated at a distance of 10 cm from the light source, while in the final surface-finishing experiment polished samples were irradiated at 254 nm for 15 min immediately before and again for 15 min after immersion in the bacterial suspension at a distance of 3 cm.

### 2.6. Densitometric Analysis of Biofilm Formation

Biofilm formation was assessed and quantified by digital image-based densitometry. Images were acquired using a ChemiDoc MP Imaging System (Bio-Rad, Hercules, CA, USA) equipped with a CCD camera and analyzed using Quantity One software version 4.6.6. (Bio-Rad, Hercules, CA, USA). The integrated optical density (IOD) of the crystal violet-stained surface was used as a quantitative measure of attached biofilm. Results are presented as natural logarithm-transformed integrated optical density values [ln(IOD)] expressed in arbitrary units (a.u.).

### 2.7. Statistical Analysis

Data are presented as mean ± standard deviation (SD). Preliminary tests of PMMA coating effect were performed using three technical and three analytical replicates for all growth and biofilm indices followed. The final experiment of PMMA surface treatment and of UV activation and of surface characterization data were obtained from three independent biological replicates (n = 3), each measured in triplicate (technical replicates). For densitometric analysis, biofilm formation was quantified by measuring the integrated optical density (IOD) of crystal violet-stained surfaces. Two biological replicates, each with two technical replicates, were analyzed. Technical replicates were averaged, and IOD values were natural log-transformed [ln(IOD)] prior to statistical analysis. A two-way ANOVA followed by Tukey’s HSD post hoc test was performed in Python using the statsmodels library (v0.14.0). Statistical significance was set at *p* < 0.05. Data are presented as ln(IOD) ± standard deviation (SD), where IOD is expressed in arbitrary units (a.u.). Graphs were created in Microsoft Excel.

## 3. Results

### 3.1. SEM Characterization of Surface Morphology and TiO_2_ Thin-Film Coverage on PMMA Substrates

**Figure 1:**
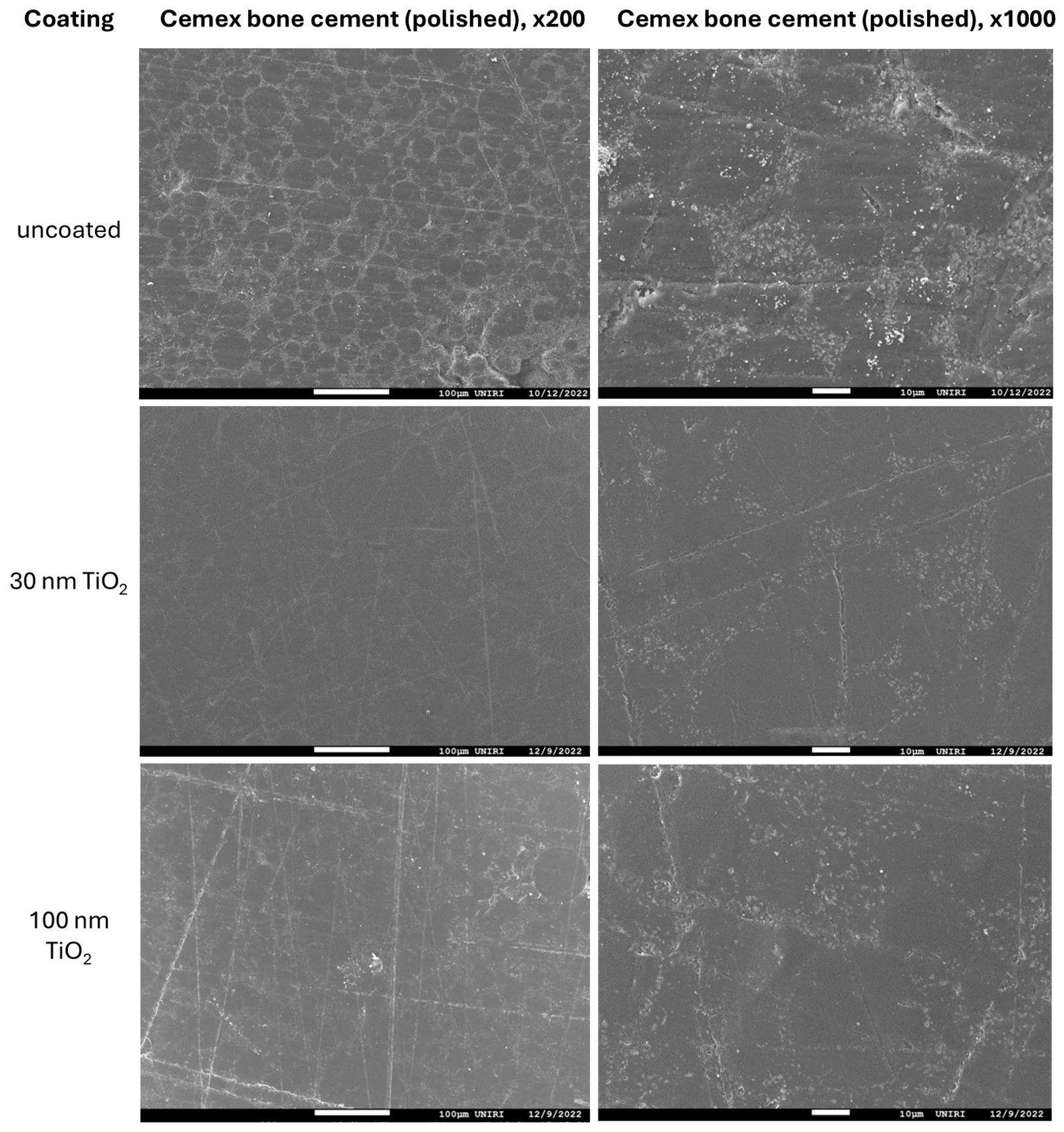
SEM images of unpolished PMMA. The first row presents images of the sample without a TiO_2_ thin film, the second row shows the sample coated with a 30 nm TiO_2_, and the third row shows the sample with a 100 nm TiO_2_ coating, both synthetized at 80 °C. Cemex bone cement was Cemex System by Tecres S.p.A., Orthopaedic Bone Cement, Verona, Italy. The left column contains images taken at 200× magnification, while the right column shows the corresponding surfaces at 1000× magnification. The images were acquired at an accelerating voltage of 10 kV, with a working distance of 10 mm, using a secondary electron detector (JEOL Ltd., Tokyo, Japan).

The surface morphology of PMMA and TiO_2_-coated samples was examined by SEM using a JEOL JSM-7800F microscope (JEOL Ltd., Tokyo, Japan) prior to microbiological testing. PMMA specimen were sputter-coated with a thin Au/Pd layer to prevent surface charging and imaged with acceleration voltage of 8 kV and 10 kV. SEM analysis was performed to evaluate surface topography and the homogeneity of the TiO_2_ coating. SEM analysis revealed differences in the surface morphology of PMMA specimen depending on TiO_2_ coating thickness (Figure 1).

**Figure 2:**
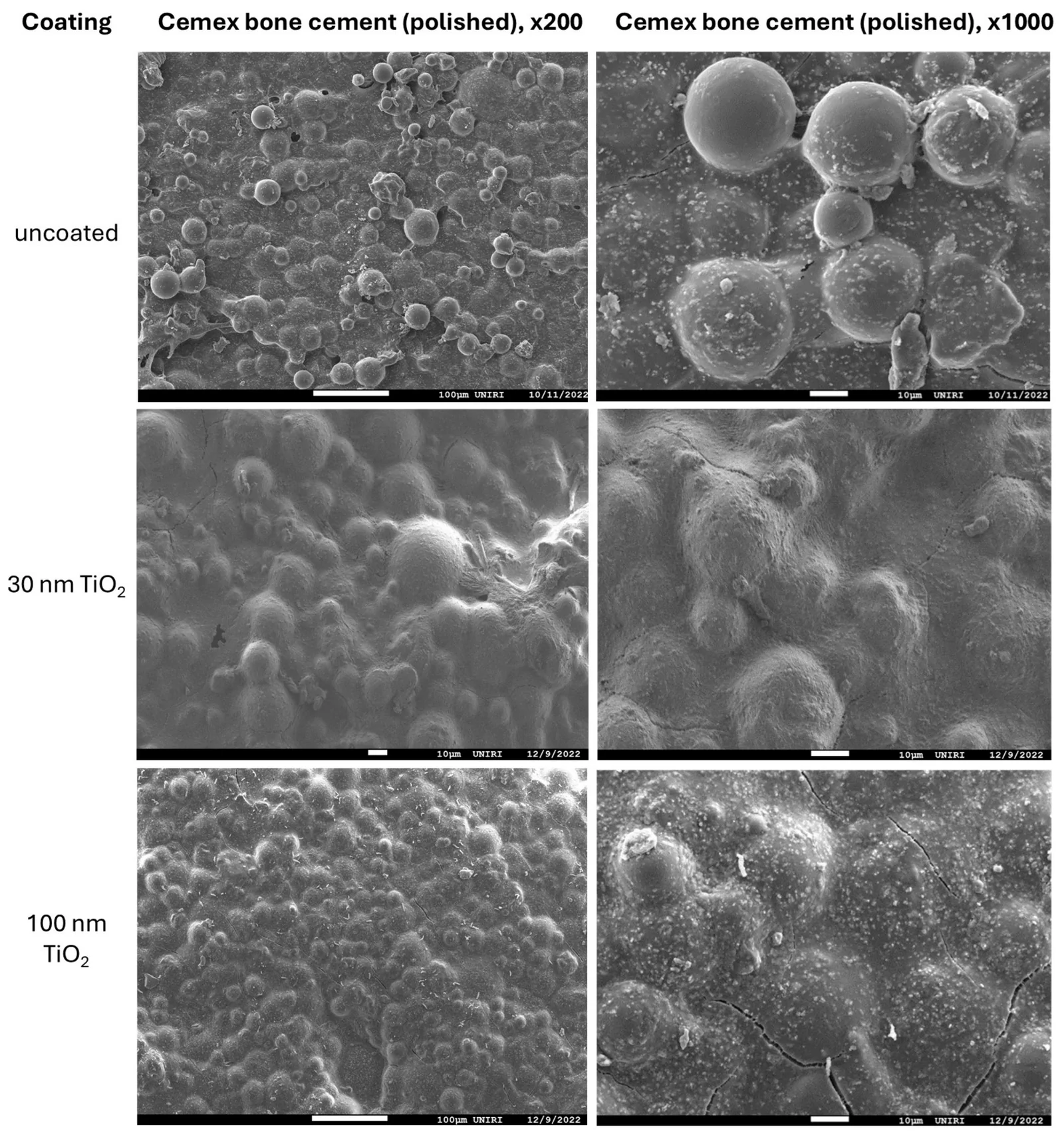
SEM micrographs of bone cement (Cemex) PMMA surfaces: uncoated (**top row**), 30 nm TiO_2_ (**middle**), and 100 nm TiO_2_ (**bottom**). Cemex bone cement was Cemex System by Tecres S.p.A., Orthopaedic Bone Cement, Verona, Italy. Images were acquired at 200× (**left**) and 1000× (**right**) using a JEOL JSM-7800F at 10 kV, 10 mm working distance, with a secondary electron detector (JEOL Ltd., Tokyo, Japan).

The surface morphology of unpolished PMMA at 200× and 1000× magnification exhibits pronounced roughness with irregular topographical features characteristic of untreated polymer substrates. Numerous spherical and semi-spherical particles of varying sizes are distributed across the surface, forming clusters in some areas while remaining isolated in others. The underlying PMMA texture is clearly visible, indicating the absence of any continuous coating or surface modification. After deposition of a 30 nm TiO_2_ thin film, the surface appears more uniform, with slight smoothing of the underlying PMMA features, although the original texture remains partially visible. In contrast, the 100 nm TiO_2_ coating produces a noticeably more homogeneous and continuous surface, with reduced apparent roughness and fewer visible surface irregularities. SEM observations suggest that increasing the TiO_2_ thickness improves coating continuity and surface coverage, although quantitative roughness measurements were not performed (Figure 2).

In comparison, the uncoated surface of polished samples displays a rough and heterogeneous morphology with visible cracks and irregularities (Figure 2). It has to be noted that the 30 nm TiO_2_ layer partially smooths the surface while still allowing the underlying structure to remain visible, indicating incomplete coverage. The 100 nm TiO_2_ coating produces a more uniform and continuous film, reducing the prominence of surface defects and resulting in a noticeably more homogeneous morphology. Cross-sectional SEM characterization of ALD-deposited TiO_2_ thin films with different thickness (30 nm and 100 nm) on Si substrate are shown in Figure S1.

Based on previous characterization by our group of TiO_2_ films deposited under identical ALD conditions (same reactor, precursors, deposition temperatures, number of cycles) [21], TiO_2_ deposited at 225 °C exhibited GIXRD diffraction peaks characteristic of crystalline anatase, whereas TiO_2_ deposited at 200 °C remained amorphous. Therefore, in the present study, TiO_2_ films deposited at 230 °C will certainly correspond to crystalline anatase, whereas the 80 °C films undoubtedly remain amorphous. 

### 3.2. Atomic Force Microscopy (AFM) Analysis of PMMA Surface Topography

**Figure 3:**
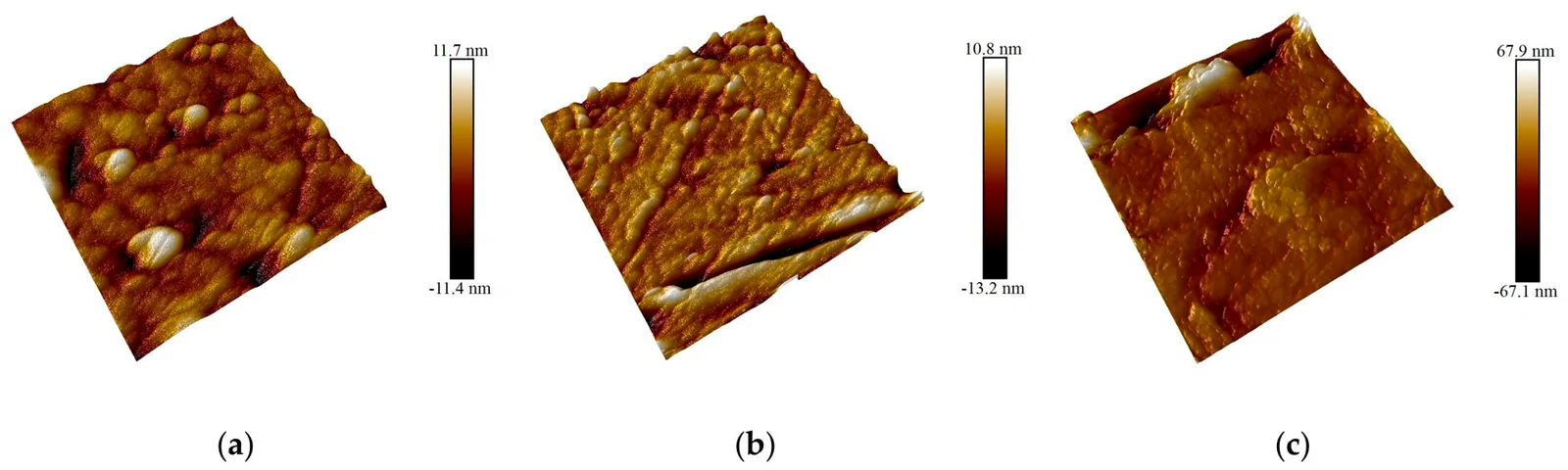
3D visualizations of 1 × 1 µm^2^ scanned topologies of three analyzed samples: (**a**) unpolished surface, (**b**) polished surface, and (**c**) polished surface coated with TiO_2_ thin film. The scans were made with scan sizes of 1 µm^2^ with 512 scan lines, with three repeated scans, each with 512 data points acquired per line.

The surface topology scans of the analyzed samples are obtained by using the atomic force microscope Bruker Dimension Icon in tapping mode. Tapping mode was used to obtain a high-resolution scan of surface detail by using a Bruker SNL-10 type D (low stiffness) silicon nitride cantilever with silicon tip of 6 nm tip radius determined via Bruker’s proprietary method from scanning a Ti tip characterizer sample. The cantilever’s dynamic and mechanical properties such as natural frequency and normal stiffness are obtained by using the thermal tune method where the thermal noise spectra of the cantilever were measured and fitted to a Lorentzian harmonic oscillator model in air which corresponded to 24 kHz, while the stiffness was obtained by determination of the slope of the linear part of the force-distance curve obtained by ramping a hard sapphire glass sample. The obtained calibration allows the usage of minimal contact force for the measurement which has minimal impact on the surface of the sample, and thus, the average used force was 3.5 pN ± 5%. The scans, shown in Figure 3, were made with scan sizes of 1 µm^2^ with 512 scan lines, with three repeated scans, each with 512 data points acquired per line. The obtained data was processed to obtain the values of surface roughness parameters, shown in Table 1, after tilt and bow corrections using the proprietary Bruker Nasoscope Analysis software (v 1.50).

The surface topology scans show that the unpolished sample exhibits larger grain sizes with height range from cca ± 11 nm, while the polished surface, albeit showing finer structural surface topology, suffers from unavoidable streaks and surface damage due to the used polishing process. Finally, the polished and subsequently coated ALD-synthesized TiO_2_ thin-film surface shows typical surface characteristics of amorphous TiO_2_ layers with lower local roughness but higher global height ranging from ±67 nm.

As observed from 3D surface scans, the standard surface roughness parameters shown in Table 1 confirm with quantitative values that the unpolished surface has the lowest roughness, with an average value of Ra of 1.58 nm, while the polished surface, due to the polishing process, demonstrates higher roughness values of about 0.5 nm but more than 50% higher maximal value of surface roughness of 36.2 nm when compared to 19.83 nm on the unpolished surface, which is taking into account the streak-shaped trenches, i.e., mechanical damage made to the surface due to polishing process used. And finally, the polished and subsequently coated surface shows the highest values of surface roughness parameters, with an average value of 14.6 nm, which is around 10 times more than the polished and unpolished counterparts, which is even more evident in the Rmax parameter value, where large TiO_2_ grains contributed to a high value of maximum roughness of 298 nm when compared to polished and unpolished samples.

### 3.3. Microbiology

Comprehensive microbiological testing was performed to evaluate the effects of TiO_2_ coating, effect of TiO_2_ deposition temperature, UV activation of TiO_2_-coated surfaces, and surface polishing state on biofilm formation across four experimental groups (unmodified PMMA, TiO_2_-coated PMMA, temperature-varied coatings, and surface-treated/UV-activated samples) by the use of two bacterial strains, namely *S. aureus* and *P. aeruginosa*. All data are presented in the Supplementary Materials. The only robust changes were observed in the results presented and statistically analyzed in the following section.

The effect of TiO_2_ coating on biofilm formation and planktonic growth of *P. aeruginosa* and *S. aureus* on PMMA surfaces was assessed by quantitative analysis of optical density. The assays were performed with three technical replicates each with three analytical replicates to describe assay repeatability of procedural manipulations and of spectroscopic measurements, respectively. The scanning coefficient of variation (CV%) averaged 7.48% (SD 7.26%), with a total range of 0.36–17.61% (min–max) and technical variability averaged 6.85% (SD 5.42%), with a total range of 0.44–15.38% (min–max) for planktonic growth OD600 results. For detached biofilm, the scanning CV% averaged 4.25% (SD 2.89%), with a total range of 0.67–10.63% (min–max), and technical CV% averaged 7.09% (SD 5.87%), with a total range of 1.55–15.28% (min–max) in OD600 results. Crystal violet-based assay showed the scanning CV% averaged 1.76% (SD 1.40%), with a total range of 0.18–4.1% (min–max), and technical CV% averaged 27.61% (SD 10.47%), with a total range of 17.82–40.88% (min–max) in OD590 ratio results. The results of the three assays are shown in Table 2.

**Figure 4:**
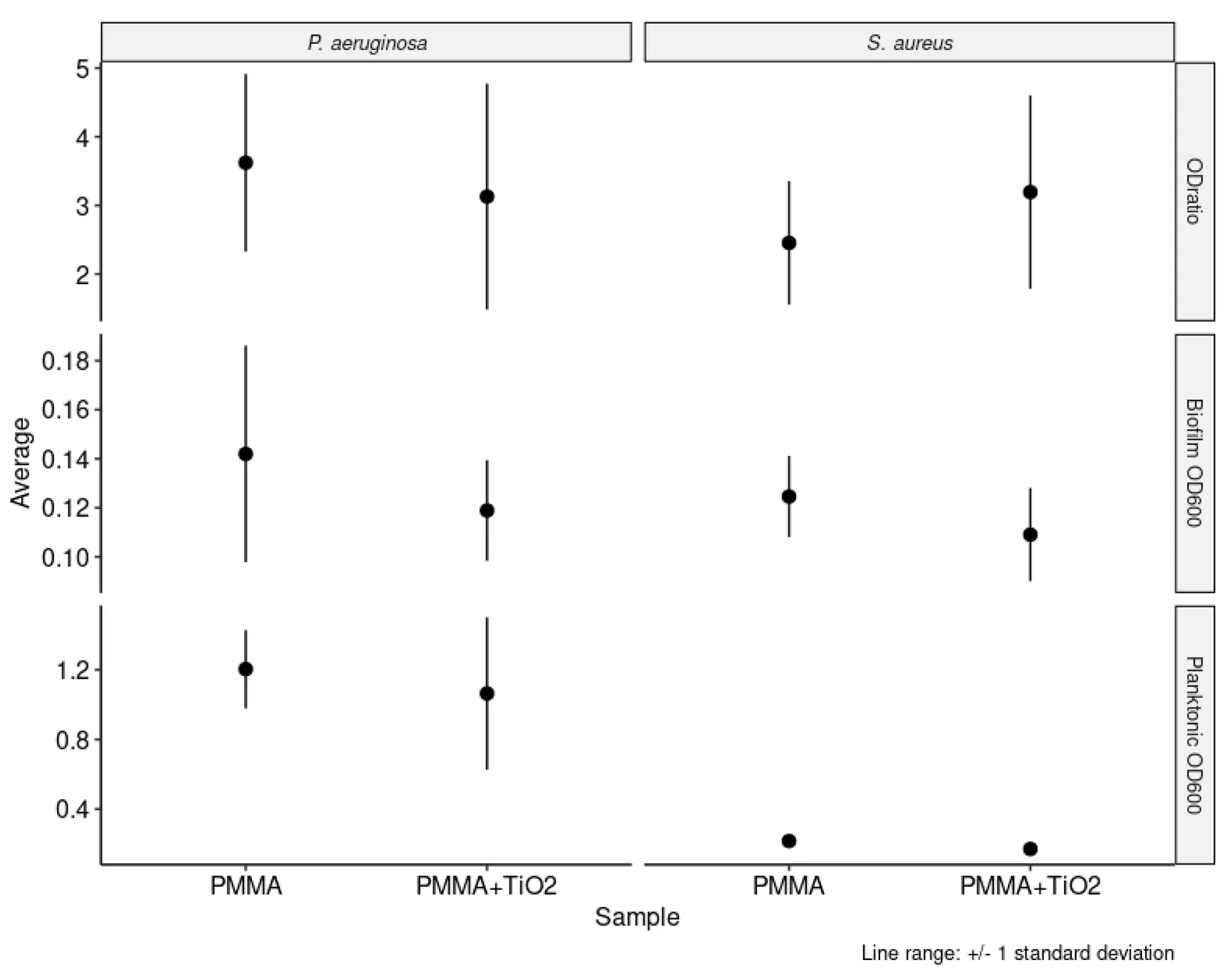
OD590 ratio representing biofilm formation based on crystal violet assay (upper row), the middle row biofilm biomass based on OD600, and the lower row planktonic growth based on OD600. Data are presented as bars denoting average points ± standard deviation of three independent experiments.

Comparison of planktonic growth, detached biofilm and OD590 ratio showed that TiO_2_ coating exhibited lower, though not significant (*p* = 0.09), planktonic growth of *S. aureus* than unpolished uncoated PMMA, as well as higher, though not significant, detached biofilm (*p* = 0.07) and OD590 ratio (*p* = 0.3) on TiO_2_-coated unpolished PMMA than on uncoated unpolished PMMA. In contrast, *P. aeruginosa* showed slightly higher planktonic growth, detached biofilm and OD590 ratio on coated unpolished PMMA (Figure 4), with only significantly higher detached biofilm results (*p* = 0.04). After this evaluation we have introduced the PMMA polishing step before amorphous TiO2 layering.

**Figure 5:**
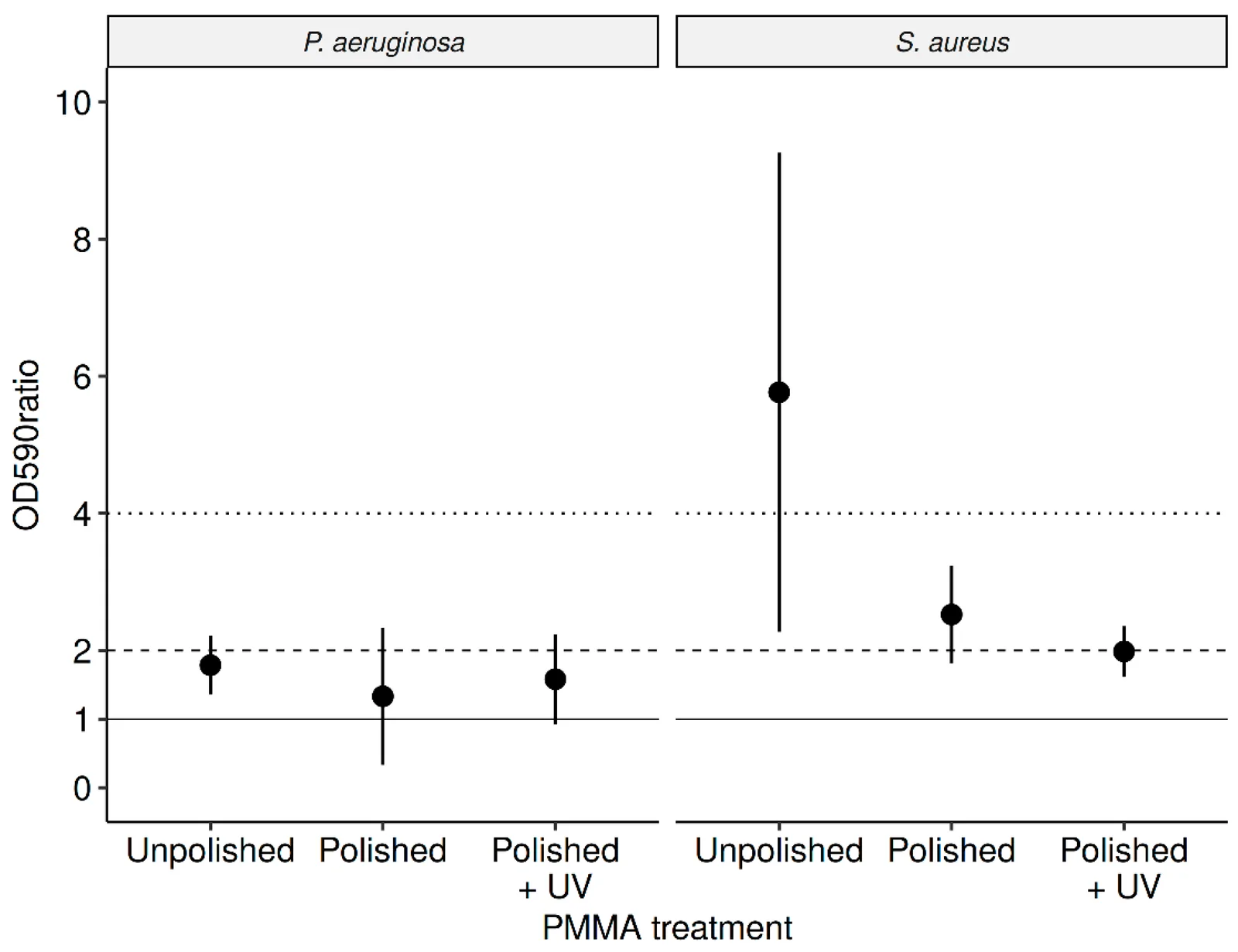
Biofilm formation trends on tested PMMA surface types before and after UV irradiation, expressed as the OD590 ratio determined by the crystal violet assay. The reproducibility of the assay showed biological variability averaged 41.39% (SD 22.36) with a total range of 18.69–75.19% (min–max) in OD590 ratio results.

The effect of surface finishing and UV treatment on biofilm formation on TiO_2_-coated PMMA was also assessed, and the results are presented in Figure 5. This experiment was performed with three biological replicates (experiment on different days) each with three technical replicates (two technical replicates from the last day were used for densitometric studies) and only used crystal violet-stained biofilm measurements. The reproducibility of the assay showed biological variability averaged 41.39% (SD 22.36) with a total range of 18.69–75.19% (min–max) in OD590 ratio results.

### 3.4. Densitometric Evaluation of Surface-Associated Biofilm Biomass

Biofilm formation was assessed and quantified by digital image-based densitometry. Images were acquired using a ChemiDoc MP Imaging System (Bio-Rad, Hercules, CA, USA) equipped with a CCD camera, and raw signal intensity values were obtained by analyzing the captured images using Quantity One software. The integrated optical density (IOD) of the crystal violet-stained surface was used as a quantitative measure of attached biofilm and is expressed in arbitrary units (a.u.). IOD values were natural log-transformed [ln(IOD)] prior to statistical analysis.

**Figure 6:**
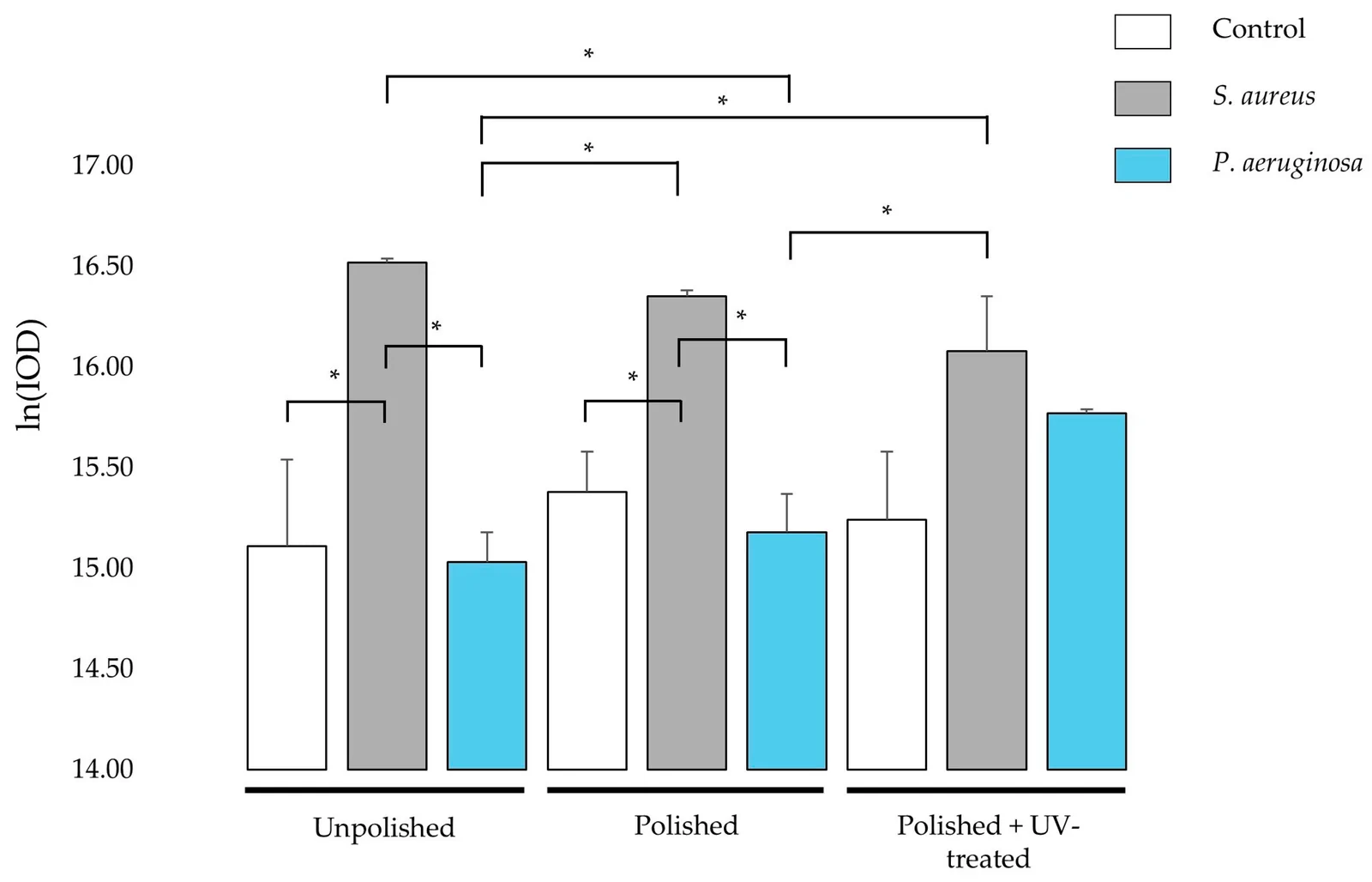
Integrated optical density (IOD) of crystal violet-stained biofilms on TiO_2_-coated PMMA surfaces with different surface treatments. Values are presented as ln(IOD) ± SD (IOD expressed in arbitrary units, a.u.). Statistical significance (*p* < 0.05) is indicated by an asterisk.

Quantitative densitometric analysis revealed differences in biofilm biomass depending on surface treatment and bacterial species (Table 3). For *S. aureus*, the highest biofilm formation was observed on unpolished PMMA surfaces (16.52 ± 0.02), followed by polished (16.35 ± 0.03) and polished + UV-treated samples (16.08 ± 0.27), indicating an overall reduction in biofilm biomass after surface modification (Table 3). In contrast, *P. aeruginosa* showed lower biofilm formation on unpolished (15.03 ± 0.15) and polished surfaces (15.18 ± 0.19), while a marked increase was observed on polished + UV-treated samples (15.77 ± 0.02). Overall, *S. aureus* exhibited higher ln(IOD) values compared to *P. aeruginosa* across all experimental conditions, except in UV-treated samples where the difference between species was reduced (Figure 6). This difference might be related to species-specific adhesion mechanisms. Unlike *S. aureus*, which relies predominantly on passive attachment to surface irregularities, *P. aeruginosa* possesses active motility systems and rapidly produces extracellular polymeric substances, enabling adaptation to a broader range of surface conditions. UV treatment may have also altered surface physicochemical properties, such as surface energy or wettability, in a manner that favored initial attachment of *P. aeruginosa*.

### 3.5. SEM Analysis of Surface Morphology and Biofilm After Crystal Violet Staining

**Figure 7:**
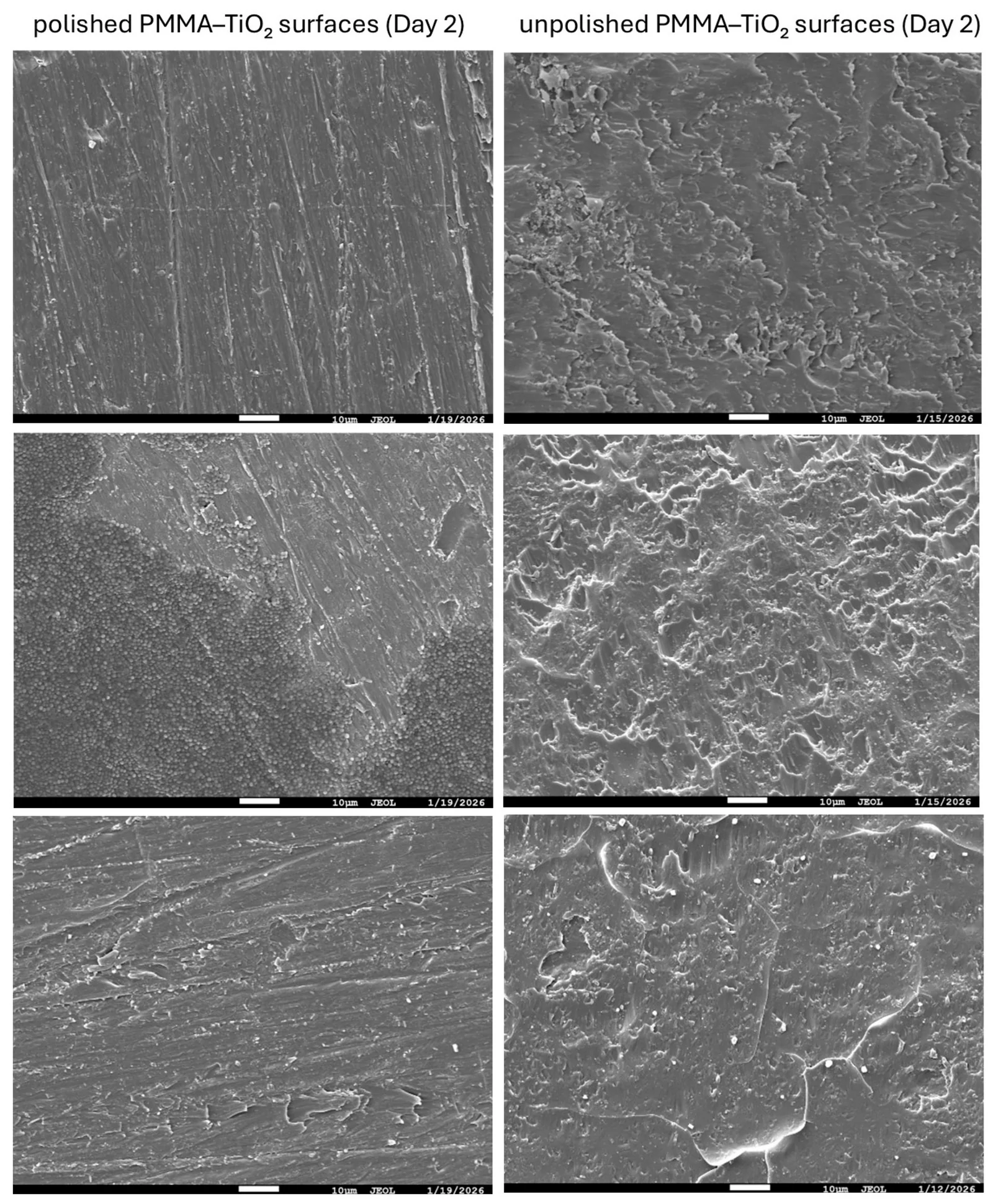
SEM micrographs of polished and unpolished PMMA–TiO_2_ surfaces (Day 2) under control conditions and after exposure to *S. aureus* and *P. aeruginosa*, showing two surface types (JEOL JSM-7800F; 1000×).

Following crystal violet staining, the surfaces were finally examined by SEM at 1000× magnification to visualize overall surface appearance and morphology. A comparison between the unpolished and polished PMMA–TiO_2_ surfaces clearly demonstrates the effect of mechanical polishing on the composite morphology. SEM image of the polished PMMA–TiO_2_ composite surface reveals a predominantly smooth morphology and more uniform appearance. There are visible polishing-induced grooves oriented mainly in one direction. These elongated, parallel lines are characteristic of mechanical polishing and indicate material removal along a uniform path. Also, localized micro-scratches and small irregularities are visible. At this magnification (×1000), no prominent particle agglomerates or surface defects are observed, so polishing produces a relatively homogeneous surface. SEM image of the unpolished PMMA–TiO_2_ composite surface shows a highly irregular morphology, dominated by pronounced roughness and heterogeneous topographic features. Local agglomerates or protrusions are visible in several areas. Such morphology indicates that the material retains its original processing-related texture (Figure 7).

Surface topography has a pronounced effect on the adhesion behavior of *S. aureus*. The SEM image of the polished PMMA–TiO_2_ surface exposed to *S. aureus* shows distinct differences in bacterial coverage across the surface. Dense clusters of spherical bacterial cells (biofilm) occupy the rougher, micro-textured region of the sample. The polished surface area exhibits significantly lower bacterial adhesion. The reduced roughness and presence of linear polishing grooves limit the number of available attachment sites and decrease mechanical interlocking between bacterial cells and the substrate. As a result, initial adhesion is weaker and spatially restricted. The unpolished PMMA–TiO_2_ surface is characterized by high roughness, irregular protrusions and deep microcavities. The surface exhibits numerous microscale depressions and protrusions that act as attachment sites for bacterial cells. These micro-areas help cells settle, stay protected, and gradually form larger colonies. When comparing the unpolished and polished PMMA–TiO_2_ surfaces, the influence of surface topography on *S. aureus* adhesion becomes highly evident. The polished surface exhibits a smoother morphology with linear polishing grooves. Bacterial coverage is significantly lower on the polished regions, and *S. aureus* appears more sporadically distributed, with cluster formation occurring primarily on areas that retain slight irregularities. SEM images of the polished PMMA–TiO_2_ surface exposed to *P. aeruginosa* show a predominantly smooth morphology with parallel polishing-induced grooves characteristic of mechanically finished polymer composites. No significant bacterial adhesion is observed across the examined area. The absence of rod-shaped *P. aeruginosa* cells and the lack of biofilm structures indicate that the polished, low-roughness surface does not provide favorable attachment sites for bacterial cells. The uniform surface topography therefore appears to inhibit initial adhesion and colonization of *P. aeruginosa* under the tested conditions. Unlike the polished specimen, the unpolished PMMA–TiO_2_ surface after exposure to *P. aeruginosa* shows numerous surface imperfections and microcavities. Only a small number of isolated particulate features are visible on the surface, but no distinct rod-shaped *P. aeruginosa* cells or biofilm-like structures can be identified at this magnification. The absence of significant bacterial coverage indicates that the rough, heterogeneous texture did not promote pronounced colonization under the tested conditions, and that adhesion remained limited to sparse, localized sites not forming larger aggregates. Overall, *S. aureus* demonstrates moderate adhesion on polished surfaces, localized mainly along polishing grooves and strong adhesion and high surface coverage on unpolished surfaces. This is due to the small size and spherical morphology, which enable attachment within even shallow or irregular microcavities. *P. aeruginosa*, conversely, demonstrates very low adhesion on both surface types.

## 4. Discussion

The herein presented findings are in line with recent research on the antimicrobial performance of amorphous TiO_2_-based coatings. In a recent review paper by Melo-Soares et al. it has been assessed that material photocatalytic properties strongly depend on the size, crystallinity, and surface morphology [22]. Our current results also support evidence pointing to TiO_2_ not only as an intrinsically antibacterial material, but more as a surface-modifying agent whose biological performance is mediated by morphology-dependent interactions. Our study assessed, indeed, how ALD of TiO_2_ modified the surface of PMMA and how the resulting micro- and nanotopography influenced bacterial adhesion under UV illumination. Surface morphology emerged as a dominant determinant of bacterial adhesion, particularly for *Staphylococcus aureus*. A major finding of this work is that TiO_2_ deposition substantially altered PMMA surface topography. Although ALD is generally associated with conformal and homogeneous coatings, AFM analysis demonstrated that coating deposition produced a pronounced increase in roughness-related parameters. The coated surface showed markedly higher Ra, Rq, and Rmax values than both unpolished and polished PMMA, indicating that TiO_2_ deposition generated a heterogeneous nanoscale landscape rather than an ideally smooth film. In particular, the Rmax value of the polished and coated group confirmed the presence of pronounced vertical heterogeneity. SEM observations supported these findings by showing progressive surface coverage with increasing TiO_2_ thickness, but also persistent microstructural irregularities. Together, these data indicate that the deposited TiO_2_ film was continuous, yet morphologically complex. These topographical changes are highly relevant from a microbiological perspective. Surface roughness is a well-established regulator of bacterial attachment because micro and nanoscale irregularities can provide protected anchorage points, reduce local shear removal, and facilitate early colonization [23]. In the present study, this effect was most evident for *S. aureus*. Biofilm formation by *S. aureus* was highest on unpolished surfaces and decreased after polishing, with a further reduction after UV treatment. SEM analysis supported the crystal violet data by demonstrating dense bacterial accumulation on rougher unpolished areas, whereas polished surfaces showed much lower and more spatially restricted adhesion. These findings are in agreement with previous reports indicating that surface topography plays a key role in regulating *S. aureus* adhesion to biomaterials [24]. These results may be explained by bacterial adhesion at the nanoscale level that is governed by the balance between van der Waals forces, electrostatic interactions, and steric effects [25]. In our study, increased roughness strengthened these interactions between the surface and bacteria. In addition, nanoscale depressions physically trapped the bacteria cells which was more pronounced for the coccoid bacteria such as *S. aureus*. This species-specific nature of the response is particularly important, as bacterial adhesion has been shown to vary significantly between species depending on surface properties and mechanical cues [26]. While *S. aureus* showed a strong dependence on surface condition, *P. aeruginosa* exhibited a comparatively limited and method-dependent response to surface modification. This differential result between *S. aureus* and *P. aeruginosa* may also be due to the differences in motility and biofilm formation properties. While *P. aeruginosa* showed consistently lower biofilm formation and minor differences across the tested groups, the crystal violet assay and densitometric analysis revealed partially different trends following surface modification. The crystal violet assay pointed to a greater biofilm biomass on unpolished surfaces, whereas densitometric analysis suggested a higher degree of surface-associated staining on polished specimens. This discrepancy may be due to different parameters measured by the two methods. Crystal violet quantifies total biofilm biomass, including bacterial cells and extracellular polymeric substances (EPS), whereas densitometric analysis primarily estimates the extent and intensity of surface-associated staining. Because *P. aeruginosa* produces abundant EPS and forms structurally heterogeneous biofilms, these methods may yield results with partial disagreement. This is why the effect of surface finishing on *P. aeruginosa* should be interpreted with caution and may be method-dependent. This differential behavior compared with *S. aureus* may also be explained by species-specific differences in motility and biofilm formation. Indeed, *S. aureus* relies primarily on passive adhesion and accumulation, while *P. aeruginosa* possesses active motility mechanisms, including swimming motility, which may reduce its dependence on surface microtopography [20]. In addition, *P. aeruginosa* produces extracellular polymeric substances that facilitate surface colonization even under less favorable topographical conditions [27,28,29,30]. An additional important observation is that UV irradiation did not produce a consistent enhancement of antibacterial performance. TiO_2_ is widely recognized for its photocatalytic activity under UV light, where excitation of the semiconductor leads to the generation of reactive oxygen species capable of damaging microbial cells [31,32]. Based on this mechanism, a stronger antibacterial effect might have been expected after UV exposure [31]. However, the present results did not show a robust and universal UV-dependent reduction in bacterial adhesion or growth. For *S. aureus*, UV treatment further reduced biofilm biomass after polishing, but this effect was not sufficiently consistent across all experiments to support a strong photocatalytic interpretation. For *P. aeruginosa*, UV-associated changes were minor and did not indicate clear bactericidal enhancement. These results suggest that TiO_2_ coating alone does not guarantee antibacterial performance, as its effect is strongly modulated by surface morphology. This result is in line with published data on amorphous TiO_2_ modest photocatalytic activity [15,16,17]. Therefore, reliance on UV-activated antibacterial mechanisms alone in this case is insufficient for clinical applications. One possible explanation is that the roughened coated surface may partially limit the efficiency of photocatalytic activation. Irregular micro- and nanostructures can create protected niches in which bacterial cells are less exposed to direct UV irradiation and, potentially, less accessible to short-lived reactive oxygen species generated at the surface. In other words, the same roughness that increases the specific surface area of the coating may also create a shielding or “shadowing” effect that reduces the practical antimicrobial benefit of UV activation [10]. This interpretation is consistent with the observation that polishing, which reduces morphological heterogeneity, had a more reproducible effect on *S. aureus* adhesion than UV exposure itself. The results also indicate that the antibacterial action of the tested surfaces is better described as anti-adhesive rather than directly bactericidal. In the planktonic/biofilm analysis, amorphous TiO_2_-coated surfaces reduced biofilm formation relative to planktonic growth without inducing a clear and uniform suppression of total bacterial proliferation. This is an important distinction. If the dominant mechanism were predominantly bactericidal, a more pronounced and consistent reduction in planktonic growth might be anticipated, as suggested by Pantaroto et al. in their study on the bactericidal efficiency of UV-active TiO_2_ thin films [15]. Instead, our data indicate that surface modification primarily altered bacterial–material interactions at the interface, influencing the ability of cells to attach and remain on the surface. This interpretation is further supported by the absence of evidence for a leaching-based antimicrobial mechanism.

From a materials design perspective, the present results suggest that optimizing antimicrobial effects on material surfaces requires a balance between chemical functionality and topographical control. The increase in surface roughness may lead to enhanced coating surface area and potentially improve photocatalytic activity. However, excessive roughness may on the other side promote bacterial adhesion. An optimal surface design may be directed accordingly to controlled nanoscale properties of the material that will minimize bacterial adhesion and exert favorable physicochemical properties [33]. The technological aspect of the study is also noteworthy. Deposition at low temperature is essential for polymeric substrates such as PMMA because elevated temperatures may compromise dimensional stability and material integrity [7]. The use of ALD at 80 °C therefore represents a significant practical advantage, enabling the fabrication of conformal TiO_2_ nanolayers on thermally sensitive polymers. At the same time, the present results suggest that low-temperature deposition must be optimized not only for coating continuity, but also for roughness control. In this context, future studies should systematically correlate ALD cycle number, film thickness, crystallinity, and post-treatment conditions with both surface energy and bacterial behavior. Several limitations should also be acknowledged. First, the sample size was small, and although the data revealed apparent trends, some variability was considerable, particularly in the microbiological assays. Second, the study included only two bacterial reference strains, which limits generalization to broader polymicrobial or clinically relevant oral biofilms. Third, UV activation was performed under controlled laboratory conditions that may not adequately reflect the intensity, wavelength distribution, or accessibility of light in the clinical environment where illumination is typically weaker and often obstructed by surrounding tissues and saliva limiting effective photocatalytic activation [34]. Finally, while the study clearly demonstrates the importance of topography, additional characterization of wettability, surface free energy, and TiO_2_ phase composition would help to further disentangle the relative contributions of chemical and physical surface properties.

Overall, the present study shows that the antibacterial behavior of amorphous TiO_2_-coated PMMA is not an intrinsic consequence of amorphous TiO_2_ deposition alone. Rather, it is the result of a balance between topography-driven bacterial attachment and photocatalytic potential. Under the tested conditions, roughness-related effects were especially important and, for *S. aureus*, appeared to outweigh any clear UV-induced antibacterial benefit. These findings challenge simplified assumptions regarding TiO_2_ as a universally antibacterial coating and emphasize that successful antimicrobial surface design must integrate chemistry, topography, and species-specific biological response.

## 5. Conclusions

Amorphous TiO_2_ nano-coatings deposited on PMMA by ALD induce significant micro- and nanoscale modifications of surface topography, which emerge as the primary factor governing bacterial interaction with material surfaces. The results demonstrate that these topographical changes outweigh the contribution of photocatalytic activity, as UV irradiation did not produce a consistent enhancement of antibacterial performance. Surface finishing, particularly polishing, effectively reduced morphological heterogeneity, highlighting the critical role of roughness control in modulating bacterial surface interactions. Overall, the findings show that amorphous TiO_2_ coating alone does not ensure antimicrobial efficacy, and that precise engineering of surface topography is essential for the development of functional PMMA-based biomaterials. Future research should focus on careful measurement of individual contributions to the surface chemistry and topography, i.e., varying roughness independently of coating composition. Moreover, from a microbiological point of view, the investigation of polymicrobial biofilms and dynamic flow conditions may yield more clinically relevant insights on antibacterial performance. Finally, the combination of TiO_2_ and other functional coatings, such as, for example, hydrophilic or anti-fouling layers, should be explored to overcome the limitations observed in the present study. Indeed, amorphous TiO_2_ tested herein should not be regarded as a universal antibacterial coating, but may be rather tested as a tunable surface engineering material with biological properties stemming from the interplay between its structure, chemistry, and microbial behavior.

## Figures and Tables

**Table 1:** Values of surface roughness parameters for three analyzed samples. Surface roughness parameters, including root mean square roughness (Rq), arithmetical mean roughness (Ra), and maximum roughness height (Rmax), were determined; all values are expressed in nanometers (nm) as mean ± standard deviation (SD).

Sample	Rq [nm] Mean ± SD	Rmax [nm] Mean ± SD	Ra [nm] Mean ± SD
Unpolished	2.19 ± 0.30	19.83 ± 2.67	1.58 ± 0.18
Polished	2.56 ± 0.54	36.20 ± 9.57	1.82 ± 0.36
Polished and coated	20.07 ± 4.78	298.00 ± 122.24	14.60 ± 4.59

**Table 2:** Effect of TiO_2_ coating on unpolished PMMA with a defined thickness of 70 nm deposited at a synthesis temperature of 80 °C on biofilm formation and planktonic growth of *P. aeruginosa* and *S. aureus* on unpolished PMMA surfaces (mean ± SD). OD600—planktonic bacterial growth; biofilm cells—detached biofilm quantification (OD600); OD590 ratio—biofilm biomass (crystal violet assay); SD—standard deviation.

Bacteria	Surface	Planktonic Growth	Detached Biofilm	OD590 Ratio
*S. aureus*	PMMA	0.210± 0.032	0.104 ± 0.005	3.256 ± 0.077
	PMMA + TiO_2_	0.154 ± 0.001	0.135 ± 0.021	4.239 ± 0.253
*P. aeruginosa*	PMMA	1.244 ± 0.155	0.098 ± 0.002	2.562 ± 0.837
	PMMA + TiO_2_	1.414 ± 0.096	0.111 ± 0.007	2.829 ±0.924

**Table 3:** Effect of surface polishing and UV treatment on biofilm formation on TiO_2_-coated PMMA (100 nm coating deposited at 80 °C). Values are expressed as natural logarithm-transformed integrated optical density [ln(IOD)] ± standard deviation (SD),with IOD expressed in arbitrary units (a.u.).

Bacteria	TiO_2_-Coated PMMA	ln(IOD) ± SD
Control	Unpolished	15.11 ± 0.43
	Polished	15.38 ± 0.20
	Polished + UV	15.24 ± 0.34
*S. aureus*	Unpolished	16.52 ± 0.02
	Polished	16.35 ± 0.03
	Polished + UV	16.08 ± 0.27
*P. aeruginosa*	Unpolished	15.03 ± 0.15
	Polished	15.18 ± 0.19
	Polished + UV	15.77 ± 0.02

## Data Availability

The original contributions presented in this study are included in the article and Supplementary Material. Further inquiries can be directed to the corresponding authors.

## Supplementary Materials

The following supporting information can be downloaded at: https://www.mdpi.com/article/10.3390/dj14080463/s1.
