# Supplementary material for: The Role of Surface Topography in Bacterial Adhesion to Amorphous TiO2-Coated PMMA
Source: Dent J (Basel). 2026 Jul 24;14(8):463. doi: 10.3390/dj14080463 (PMC13511210; doi:10.3390/dj14080463)
Supplement: Supplementary file 1 [file dentistry-14-00463-s001.zip › dentistry-4363496-supplementary.pdf]

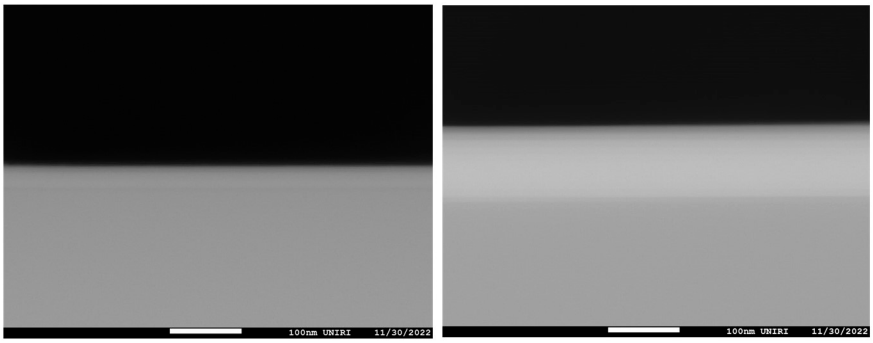

Figure S1. Control cross-sectional SEM images of ALD-deposited TiO_2 films (30 nm, left; 100 nm, right) on Si substrates, acquired at 200,000× using a JEOL JSM-7800F (12 kV, 4 mm working distance, BSE detector).

Table S1. Biofilm biomass after detachment (OD600)

Biofilm biomass determined spectrophotometrically (OD600) after 24 h of incubation, following detachment from TiO_2-coated dental PMMA (70 nm, 80 °C) prior to cell quantification by serial dilution. Results are presented as mean ± SD from three independent experiments, with CV (%) reported.

<table>
<tr><th rowspan="2"></th><th colspan="3">PMMA</th><th colspan="3">PMMA TiO_2</th><th colspan="3">Negative control</th></tr>
<tr><th>Mean</th><th>SD</th><th>CV (%)</th><th>Mean</th><th>SD</th><th>CV (%)</th><th>Mean</th><th>SD</th><th>CV (%)</th></tr>
<tr><td>S. aureus</td><td>0.10</td><td>0.01</td><td>4.8</td><td>0.14</td><td>0.02</td><td>15.28</td><td rowspan="2">0.09</td><td rowspan="2">0.01</td><td rowspan="2">13.23</td></tr>
<tr><td>P. aeruginosa</td><td>0.10</td><td>0.002</td><td>1.55</td><td>0.11</td><td>0.01</td><td>6.74</td></tr>
</table>

Table S2. Viable counts (log_{10} CFU/mL)

After 24 h of incubation, samples were UV-activated (254 nm, 10 min), followed by biofilm detachment and quantification by viable counting using tenfold serial dilutions. Data are expressed as log_{10}(CFU/mL) and presented as mean ± SD from three independent experiments, with CV (%) reported.

	PMMA			PMMA TiO_2		
	Mean	*SD*	CV (%)	Mean	*SD*	CV (%)
S. aureus	9.03	0.69	7.60	8.99	0.67	7.42
P. aeruginosa	6.73	0.25	3.64	9.68	0.81	8.37

Table S3. Biofilm formation on surface (OD590 ratio)

Biofilm formation on TiO_2-coated dental PMMA (70 nm, 80 °C) with crystal violet after 24 h of incubation, and biomass was quantified as OD590 ratio. Data are expressed as mean ± SD from three independent experiments with CV (%) reported.

	PMMA			PMMA TiO_2		
	Mean	*SD*	CV (%)	Mean	*SD*	CV (%)
S. aureus	3.26	0.08	2.35	4.24	1.25	29.56
P. aeruginosa	2.56	0.84	32.67	2.83	0.92	32.66

Table S4. Planktonic growth with/without UV (OD600)

Planktonic bacterial growth on TiO_2-coated dental PMMA (70 nm, 80°C) determined spectrophotometrically (OD600) after 24 h of incubation with UV activation at 366 nm for 1 h in a UV chamber. Data are presented as mean ± SD from three independent experiments with CV (%) also reported.

	UV Activation	PMMA			PMMA TiO_2		
		Mean	*SD*	CV (%)	Mean	*SD*	CV (%)
S. aureus	Yes	0.03	0.01	16.44	0.27	0.08	31.32
	No	0.38	0.06	15.55	0.31	0.11	34.24
P. aeruginosa	Yes	1.52	0.11	7.22	1.40	0.21	15.25
	No	1.48	0.11	7.37	1.56	0.04	2.77

Table S5. Biofilm biomass with and without UV irradiation (OD600)

Biofilm biomass determined spectrophotometrically (OD600) after 24 h of incubation, following detachment from TiO_2-coated dental PMMA (70 nm, 80 °C) surfaces and prior to cell quantification by serial dilution. Results are presented as mean ± SD from three independent experiments, with CV (%) reported.

	UV Activation	PMMA			PMMA TiO_2		
		Mean	*SD*	CV (%)	Mean	*SD*	CV (%)
S. aureus	Yes	0.11	0.02	18.34	0.12	0.01	7.78
	No	0.12	0.01	7.23	0.31	0.11	34.24
P. aeruginosa	Yes	0.10	0.01	7.42	0.10	0.002	1.89
	No	0.10	0.004	3.75	0.10	0.004	3.86

Table S6. Biofilm formation with and without UV irradiation (OD590)

Biofilm formation on TiO_2-coated dental PMMA (70 nm, 80 °C) was assessed after 24 h of incubation by crystal violet staining (OD590 ratio). Samples were UV-activated at 366 nm for 1 h immediately after incubation. Data are expressed as mean ± SD from three independent experiments, with CV (%) reported.

	UV Activation	PMMA			PMMA TiO_2		
		Mean	*SD*	CV (%)	Mean	*SD*	CV (%)

S. aureus	Yes	5.99	0.34	5.60	2.10	0.51	24.44
	No	5.37	0.55	10.17	2.81	0.15	5.47
P. aeruginosa	Yes	3.83	4.05	105.63	0.94	0.73	77.65
	No	2.21	0.85	38.49	0.66	0.13	19.57

Table S7. Planktonic growth (OD600) under different UV activation conditions

Planktonic bacterial growth on TiO_2-coated (70 nm) dental PMMA surfaces synthesized at 80 °C and 230 °C was determined spectrophotometrically (OD600) after 24 h of incubation following multiple UV activation conditions. Data are presented as mean ± SD from two independent experiments with CV (%) also reported.

UV Condition	Bacteria	80 ˚C			230 ˚C		
		Mean	SD	CV (%)	Mean	SD	CV (%)
UV-	*S. aureus*	0.17	0.55	32.16	0.11	0.03	23.74
	P. aeruginosa	1.30	0.01	0.95	1.43	0.07	4.80
366 nm, 1 h	*S. aureus*	0.10	0.02	14.42	0.13	0.002	2.21
	P. aeruginosa	1.33	0.08	6.35	1.42	0.03	2.20
366 nm, 10 min	*S. aureus*	0.18	0.04	23.79	0.19	0.01	5.53
	P. aeruginosa	1.42	0.04	7.33	1.44	0.004	0.29
254 nm, 1 h	*S. aureus*	0.12	0.001	0.95	0.21	0.02	9.88
	P. aeruginosa	1.57	0.13	8.41	1.46	0.01	0.80
254 nm, 10 min	*S. aureus*	0.21	0.04	18.77	0.16	0.01	9.80
	P. aeruginosa	1.59	0.04	2.24	1.49	0.03	2.08
UV-	*S. aureus*	0.13	0.04	30.99			
		0.11	0.03	32.49			
UV-	*P. aeruginosa*	1.44	0.06	3.84			
		1.67	0.02	1.37			
366 nm, 1 h	*S. aureus*	0.07	0.02	23.13			
	P. aeruginosa	1.60	0.02	1.22			
254 nm, 1 h	*S. aureus*	0.07	0.02	31.86			
	P. aeruginosa	1.58	0.05	3.13			

Table S8. Biofilm biomass after detachment (OD600)

Biofilm (OD600) was measured after 24 h of incubation. TiO_2-coated dental PMMA samples (70 nm, deposited at 80 °C or 230 °C) were first subjected to UV activation (254 or 366 nm for 10 min or 1 h) followed by biofilm detachment and subsequent serial dilution-based cell quantification. Data are presented as mean ± SD from three independent experiments with CV (%) also reported.

UV Condition	Bacteria	80 °C			230 °C		
		Mean	SD	CV (%)	Mean	SD	CV (%)
UV-	*S. aureus*	0.01	0.01	142.11	0.03	0.03	108.33
	P. aeruginosa	0.08	0.04	50.19	0.12	0.15	119.86
366 nm, 1 h	*S. aureus*	0.01	0.0006	10.00	0.04	0.05	137.59
	P. aeruginosa	0.18	0.16	87.63	0.02	0.02	116.19
366 nm, 10 min	*S. aureus*	0.02	0.01	47.64	0.01	0.02	141.50
	P. aeruginosa	0.09	0.0003	0.35	0.05	0.06	139.82
254 nm, 1 h	*S. aureus*	0.02	0.01	99.31	0.04	0.03	78.09
	P. aeruginosa	0.09	0.06	64.88	0.04	0.01	24.34
254 nm, 10 min	*S. aureus*	0.01	0.004	50.58	0.01	0.01	69.79
	P. aeruginosa	0.06	0.006	9.77	0.08	0.003	4.30

Table S9. Biofilm formation on surface (OD590 ratio)

Biofilm formation on TiO_2-coated (70 nm, deposited at 80 °C or 230 °C) dental PMMA was assessed by crystal violet staining after 24 h of incubation and quantified as the OD590 ratio.

UV Condition	Bacteria	80 °C			230 °C		
		Mean	SD	CV (%)	Mean	SD	CV (%)
UV-	*S. aureus*	3.58	1.88	52.45	0.82	0.27	32.59
	P. aeruginosa	6.36	0.05	0.86	4.27	0.13	3.04
366 nm, 1 h	*S. aureus*	3.31	0.48	14.38	5.09	4.80	94.34
	P. aeruginosa	5.13	0.93	18.29	11.43	0.11	0.99
366 nm, 10 min	*S. aureus*	2.63	0.45	17.31	3.09	1.87	60.59
	P. aeruginosa	3.52	0.81	23.10	12.17	1.00	9.20
254nm, 1 h	*S. aureus*	3.38	0.46	13.48	1.33	0.30	22.51
	P. aeruginosa	4.02	0.06	1.48	4.55	1.18	25.79
254nm, 10 min	*S. aureus*	2.32	1.08	46.56	1.02	0.10	10.12
	P. aeruginosa	4.16	0.09	2.09	4.60	0.43	9.24
